# GLS2 inhibition synergizes with copper to reprogram TCA cycle for cuproptosis-driven radiosensitization in esophageal cancer

**DOI:** 10.1186/s40164-025-00653-4

**Published:** 2025-04-10

**Authors:** Wang Jing, Wenhao Wang, Yi Ding, Renya Zeng, Hui Zhu, Zhichao Kang, Alei Feng, Zhe Yang

**Affiliations:** 1 Shandong Provincial Hospital Affiliated to Shandong First Medical University, Jinan, 250000 China; 2 Department of Medical Oncology, Affiliated Hospital of Shandong Second Medical University, Weifang, 261042 China; 3 Department of Radiation Oncology, Shandong Provincial Hospital, Jinan, 250000 China; 4 Department of Radiation Oncology, Shandong Cancer Hospital Affiliated to Shandong First Medical University, Jinan, 250000 China

**Keywords:** Cuproptosis, Esophageal cancer, Radiotherapy, Cancer metabolism, GLS2

## Abstract

Esophageal squamous cell carcinoma (ESCC) is notorious for its poor prognosis. In the present study, the role of glutaminase 2 (GLS2) and copper (Cu) in the radiosensitivity of ESCC was explored. Both in vitro and in vivo experiments were conducted, and the results demonstrated that the knockdown of GLS2 could suppress cell proliferation and augment the sensitivity to radiotherapy (RT). The addition of Cu influenced cell viability and radiosensitivity. Notably, under normal GLS2 expression status, exogenous Cu augmented RT sensitivity without triggering cuproptosis. Mechanistically, the suppression of GLS2 interacted with Cu to downregulate lipoic acid synthase and dihydrolipoamide S-succinyltransferase, resulting in the reduction of the activity of α-ketoglutarate dehydrogenase complex and the obstruction of the tricarboxylic acid cycle, ultimately leading to the enhancement of RT sensitivity. These findings emphasize the significance of cuproptosis in ESCC radiotherapy and provide potential directions for therapeutic strategies.

**Supplementary Information:**

The online version contains supplementary material available at 10.1186/s40164-025-00653-4.

**To the editor,**

Esophageal squamous cell carcinoma (ESCC), the predominant esophageal cancer subtype in China, has poor survival rates [1]. Radiotherapy (RT), a primary treatment for ESCC, struggles to augment radiosensitivity. Exploring novel cellular mechanisms related to cancer cell metabolism and death, especially cuproptosis, which is a copper-dependent death form that induces demise by binding acylated proteins and inhibiting the tricarboxylic acid (TCA) cycle, offers a potential solution [2–4]. Bioinformatic analyses suggest a link between cuproptosis and tumor prognosis, yet the mechanisms are unclear [5, 6]. Since modulating the TCA cycle via the copper-dependent cuproptosis pathway might impact tumor metabolism, it could be an effective strategy to enhance radiosensitivity [7–10]. Glutaminase (GLS2), which encodes glutaminase, catalyzes glutamine-to-glutamate conversion for α-ketoglutarate (α-KG) synthesis in the TCA cycle. GLS2 upregulation in esophageal cancer ferroptosis correlates with RT resistance, yet its carcinogenic mechanism remains undefined, prompting TCA cycle-focused investigation. In light of this, we hypothesized that cuproptosis-driven changes in the TCA cycle might involve critical regulatory factors. Herein, we initially present the novel finding that, with exogenous copper, inhibiting GLS2 expression suppresses the E2 component (Dihydrolipoamide S-succinyltransferase, DLST) of α-ketoglutarate dehydrogenase complex (α-KGDHC), blocks the TCA cycle consequently, and improves the radiosensitivity of ESCC.

In the present study, TCGA data indicated that GLS2 is significantly overexpressed in ESCC (Fig. 1A). By examining pre-treatment specimens from 53 ESCC patients who underwent neoadjuvant chemoradiotherapy, we found that 73.6% had high GLS2 expression, correlating with poorer pathological complete response (43.6% vs. 85.7%, *p* = 0.01; Additional file 1). GLS2 knockdown led to reduced cell proliferation, slower colony formation, impaired wound healing capacity, and enhanced RT sensitivity (Fig. 1B-E). These findings suggest that GLS2 could serve as a potential biomarker for ESCC radiotherapy. Methods and materials of this study can be found in the supplementary material.

**Fig. 1:**
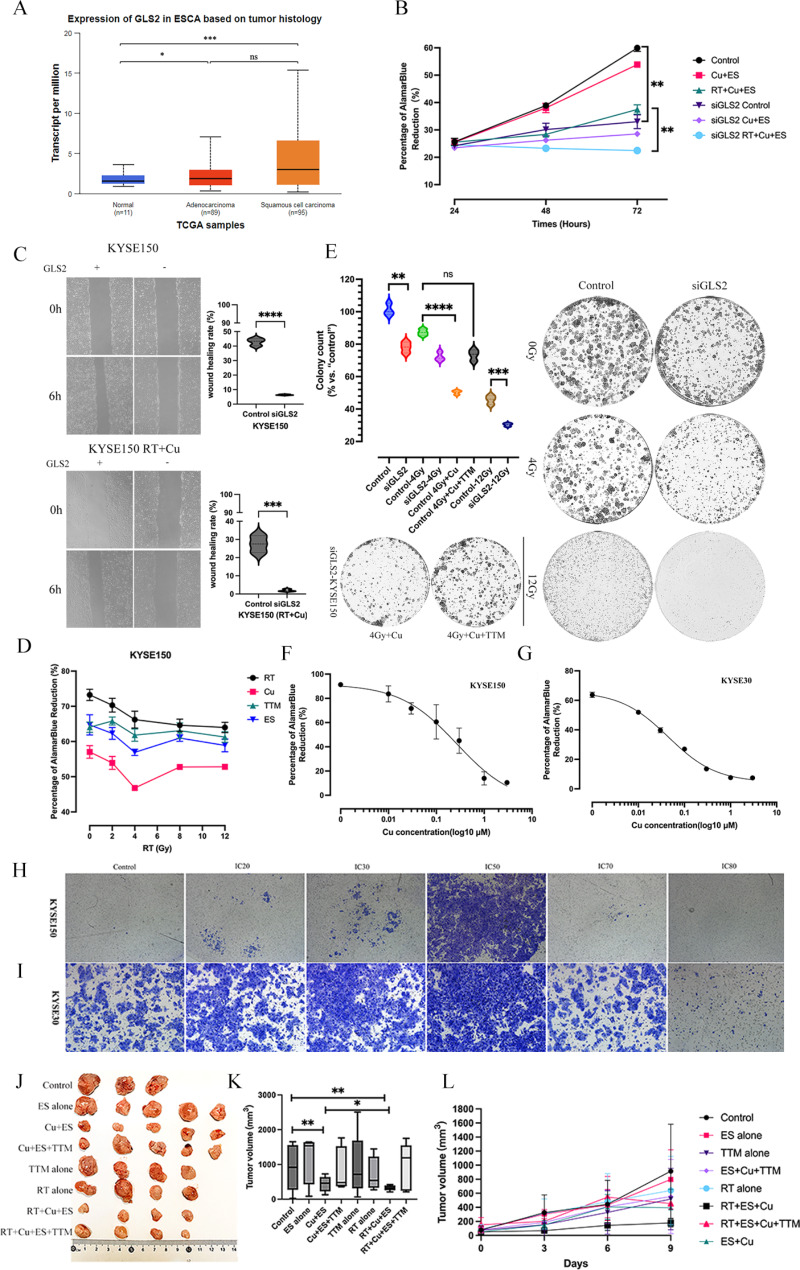
Impacts of GLS2 and Cu on ESCC radiosensitivity. (**A**) GLS2 expression in ESCC based on TCGA database. (**B**-**D**) Proliferation activity (**B**), colony count (**C**), and Wound healing (**D**) of siGLS2 KYSE150 cells and their parental cells under Cu and/ or Radiation. (**E**) Proliferation activity of KYSE150 cells treated with Cu + ES (Cu), Cu + Elesclomol (ES) + Tetrathiomolybdate (TTM), or ES alone (ES) at different RT doses. (**F**-**I**) Half-maximal inhibitory concentration (IC50) values of Cu for KYSE150 cells (**F**) and KYSE30 cells (**G**), and typical transwell images of KYSE150 cells (**H**) and KYSE30 cells (**I**) under graded concentrations of Cu. (**J**-**L**) Representative images (**J**), tumor volume (**K**) and growth curves (**L**) of KYSE150 tumors. Treatment details in vivo experimental groups: Control: Untreated mice; ES alone: Elesclomol alone; Cu + ES: Copper chelates (Cu) plus ES; Cu + ES + TTM: Cu, ES + Tetrathiomolybdate (TTM); TTM alone: Tetrathiomolybdate alone; RT alone: Radiation therapy (RT) alone; RT + Cu + ES: Combined radiation (RT + copper chelate and ES); RT + Cu + ES + TTM: Combination therapy with TTM. Cu, ES (both dose 3.5 mg/kg, intravenously) and TTM (10 mg/kg, intraperitoneally) were administered once every 3 days for a total of 3 doses (days 1, 3, and 5); RT total dose 12 Gy, 4 Gy × 3 fractions (days 1, 3, and 5). Treatment details in vitro experimental groups: Concentration: Ratio of Cu (at the IC30 concentration) vs. ES vs. TTM: 1:1:1; siGLS2: 20 pmol. RT was administrated after 4 h post-Cu treatment. (**p < 0.01, ***p < 0.001, ****p < 0.0001 by one-way ANOVA with Tukey’s HSD test). Data are mean ± SE, *n* = 3

While the significance of GLS2 in ESCC has been demonstrated, the interaction between GLS2 and external factors such as copper (Cu) in influencing cellular responses and RT-sensitivity remains to be explored. To clarify the role of the cuproptosis pathway activation as a crucial factor, we first evaluated the effect of different concentrations of Cu on cell biological behavior and RT-sensitivity. Low concentrations of Cu promoted cell viability, whereas high concentrations of Cu (> IC50) inhibited cell proliferation (Fig. 1F-I). Upon the addition of Cu to RT, a more pronounced inhibition of cell proliferation was observed in contrast to the effects of Cu alone or RT alone (Fig. 1D). Notably, this enhanced inhibitory effect could be attenuated by the Cu chelator Tetrathiomolybdate (TTM; Fig. 1. D, E). Using the KYSE150 cell line to establish tumor xenograft models, we employed treatment regimens involving RT ± Cu ± TTM (8 groups). The results indicated that the Cu treatment group, relative to the control, manifested deceleration in tumor growth and a reduction in tumor volume (Fig. 1G-L). Significantly, the RT + Cu group exhibited more remarkable outcomes, featuring a substantially slower tumor growth rate and a markedly diminished tumor volume as compared to those treated with RT alone or Cu alone. However, under normal GLS2 expression, there was no significant change in the expression of key cuproptosis proteins lipoic acid synthase (LIAS) and DLST. Those findings indicate that the addition of exogenous Cu enhanced RT sensitivity, it did not activate the cuproptosis pathway.

Given the observed enhancing effect of exogenous Cu on RT sensitivity under normal GLS2 conditions, it becomes crucial to further investigate the specific mechanisms by which GLS2 inhibition interacts with the Cu-mediated effects to enhance RT sensitivity. Initially, we hypothesized that the increased sensitivity to RT + Cu following GLS2 inhibition could be attributed to two potential mechanisms: either a reduction in α-KG levels leading to TCA cycle inhibition, or an increase in reactive oxygen species (ROS), but neither was observed (Fig. 2A-C). TCGA data indicated that GLS2 expression was positively correlated with LIAS expression-a key cuproptosis protein (Fig. 2D). Western blot indicated that LIAS expression was significantly downregulated with GLS2 knockdown, and subsequently, the expression of DLST was also markedly reduced (Fig. 2E). Further evaluation of DLST expression changes in cells with GLS2 knockdown receiving RT + Cu treatment showed significantly lower expressions of both DLST and LIAS compared to the GLS2 + RT + Cu treatment group (Fig. 2F). Since DLST is the most crucial E2 component of α-KGDHC, the activity of α-KGDHC was assessed and revealed which was significantly lower in the knockdown-GLS2 group than the parental cell group receiving RT + Cu treatment (Fig. 2G). The addition of monomethyl succinate was sufficient to rescue the defect in cell growth resulting from GLS2 knockdown in ESCC cells receiving RT + Cu treatment (Fig. 2H).

**Fig. 2:**
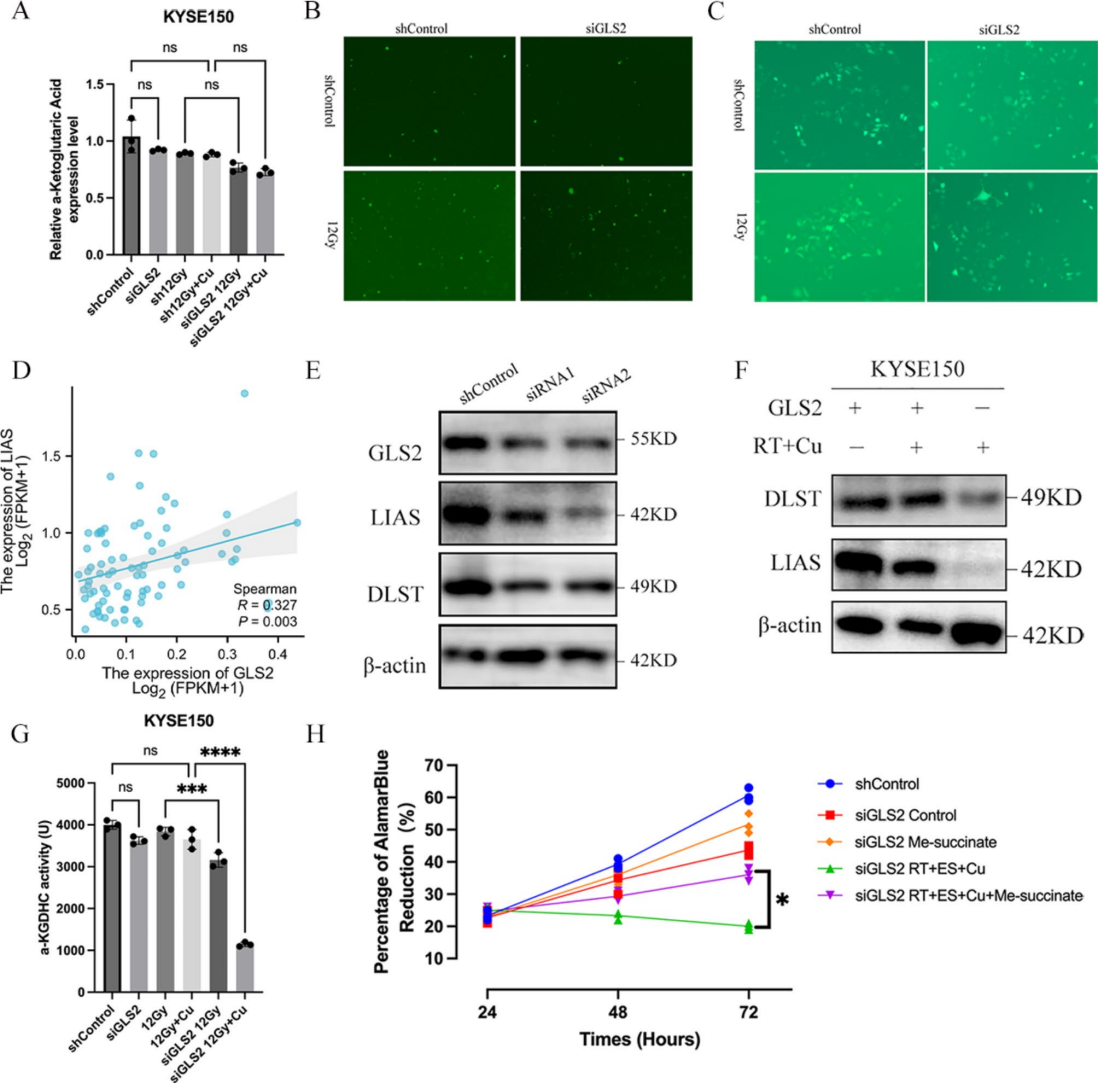
The mechanism of GLS2 promoting radiosensitivity via cuproptosis in the presence of Cu. (**A**) α-KG levels in GLS2-knockdown KYSE150 cells and parental cells treated with RT (12 Gy) plus copper. (**B**, **C**) ROS levels in parental KYSE150 cells (**B**) or KYSE30 cells (**C**) and corresponding siGLS2 cells treated with radiation (12 Gy) or not. (**D**) The correlation of GLS2 and LIAS in ESCC. (**E**, **F**) DLST and LIAS protein expression levels decreased following GLS2 knockdown (**E**) and in siGLS2 cells treated with RT (12 Gy) + Cu (F; +, normal GLS2 expression; -, GLS2 knockdown). (**G**) α-KGDHC activity was detected 24 h after radiation (12 Gy) ± Cu in KYSE150 cells with GLS2 knockdown and the parental cells. (**H**) Succinate rescues KYSE150 cells from slowed cellular growth induced by GLS2 knockdown and RT + Cu. (***p < 0.001, ****p < 0.0001 by one-way ANOVA with Tukey’s HSD test). Data are mean ± SE, *n* = 3

In conclusion, unlike its role in ferroptosis [11], GLS2 modulates cuproptosis via metabolic rewiring rather than antioxidant depletion. GLS2 modulation, in the presence of exogenous Cu, leads to an enhancement in RT sensitivity in ESCC through the downregulation of LIAS and DLST, the reduction of α-KGDHC activity, and the blockade of the TCA cycle. We do not exclude the existence of other latent mechanisms by which Cu contributes to the radiosensitization of esophageal cancer. However, copper-dependent cuproptosis is likely to be an effective pathway for achieving radiosensitization. These findings underscore the vital role of cuproptosis in ESCC and pave the way for innovative treatment strategies that exploit cancer cell metabolism. This study leaves numerous unresolved questions, particularly regarding the applicability of its experimental conditions to fundamental research on other tumor types. Nevertheless, our work established a robust framework for understanding cuproptosis in ESCC. Future studies should focus on its clinical translation and targeted therapeutic strategies.

## Electronic supplementary material

Below is the link to the electronic supplementary material.

Supplementary Material 1

Supplementary Material 2

## Data Availability

No datasets were generated or analysed during the current study.

## Abbreviations

ESCC: Esophageal squamous cell carcinoma
RT: Radiotherapy
TCA: Tricarboxylic acid
GLS2: Glutaminase 2
DLST: Dihydrolipoamide S-succinyltransferase
α-KGDHC: α-ketoglutarate dehydrogenase complex
α-KG: α-ketoglutarate
Cu,Copper: Copper chloride
ES: Elesclomol
TTM: Tetrathiomolybdate
LIAS: Lipoic acid synthase
