# Supplementary material for: GLS2 inhibition synergizes with copper to reprogram TCA cycle for cuproptosis-driven radiosensitization in esophageal cancer
Source: Exp Hematol Oncol. 2025 Apr 10;14:55. doi: 10.1186/s40164-025-00653-4 (PMC11983968; doi:10.1186/s40164-025-00653-4)
Supplement: Supplementary file 1 — Supplementary Material 1 [file 40164_2025_653_MOESM1_ESM.docx]

Table 1. GLS2 expression and its correlation with pathological response rate (pCR).

|  | GLS2 expression (N, %) | | *p* value |
| --- | --- | --- | --- |
|  | High | Low |  |
| pCR | 17 (43.6) | 12 (85.7) | 0.01 |
| Non-pCR | 22 (56.4) | 2 (14.3) |  |
