# Supplementary material for: GLS2 inhibition synergizes with copper to reprogram TCA cycle for cuproptosis-driven radiosensitization in esophageal cancer
Source: Exp Hematol Oncol. 2025 Apr 10;14:55. doi: 10.1186/s40164-025-00653-4 (PMC11983968; doi:10.1186/s40164-025-00653-4)
Supplement: Supplementary file 2 — Supplementary Material 2 [file 40164_2025_653_MOESM2_ESM.docx]

# **Materials and Methods**

**Patients and Specimens**

The medical records of patients with histopathologically confirmed esophageal squamous cell carcinoma who underwent neoadjuvant radiochemotherapy at Shandong Cancer Hospital from December 2020 to July 2022 were retrospectively reviewed. The chemotherapy was based on platinum-based Taxol, and the radiation therapy was delivered at a dosage ranging from 40 Gy - 50.4 Gy in fractions of 1.8 - 2.0 Gy each time for a total of 20 - 28 fractionations. A total of 53 patients were analyzed, and 159 specimens were obtained from those patients. Informed consent was waived since the data were collected retrospectively. The study involving human subjects was approved by the institutional review board of Shandong Provincial Hospital (No. 2023-595) and was performed in accordance with the Declaration of Helsinki.

**Cell Culture**

The human cell lines KYSE-150 and KYSE-30 were purchased from the Cell Bank of the Chinese Academy of Sciences (Shanghai, China). All the cells were cultured at Roswell Park Memorial Institute (RPMI) 1640 (Gibco) supplemented with 10% fetal bovine serum (FBS, Gibco) and 1% penicillin/streptomycin (HyClone) at 37℃ under 95% air and 5% CO_2_.

**Immunohistochemistry (IHC)**

The paraffin-embedded samples obtained from biopsies prior to neoadjuvant therapy were retrieved for the determination of target protein expression via IHC. LIAS (1:50, Proteintech), DLST (1:50, ABclonal), and GLS2 (1:200, Origene) were used in this study, and secondary antibodies (HRP-conjugated AffiniPure Goat Anti-Rabbit IgG, Proteintech) were applied. An isotype-matched rabbit IgG antibody served as a control to rule out the possibility of nonspecific or false positive staining in each sample stained for target proteins.

The staining intensity and proportion for each slide were independently evaluated by at least two pathologists who were blinded to the clinical outcomes. The color gradations were light brown, brown, and dark brown to black, respectively. The intensity scores were assigned as follows: 0 for no staining, 1+ for weak staining, 2+ for moderate staining, and 3+ for strong staining. The histoscore was determined via the following formula: Histoscore = (% weak [1+] × 1) + (% moderate [2+] × 2) + (% strong [3+] × 3), where the percentage refers to the proportion of tumor cells exhibiting weak staining relative to the total population of tumor cells.

**Cell Viability Assay**

The cells were seeded at 3000 per well in 96-well plates and incubated for 24 h prior to the administration of various treatments as per the research protocol. Alamar blue agent (Thermo Fisher Scientific) was added to the medium 2 - 6 hours before the viability was assessed by measuring the optical density at excitation wavelengths of 530 - 560 nm and emission wavelengths of 590 nm. Copper chloride was purchased from MACKLIN (Shanghai Macklin Biochemical Technology Co., Ltd), diluted to 10 mM with double-distilled water, and then serially diluted to 1 nM, 10 nM, 50 nM, 100 nM, 500 nM, and 1 μM or other working concentrations needed in this study. Elesclomol (ES, MedChemExpress) was utilized as a carrier for copper chloride, and the two were mixed at a 1:1 ratio in subsequent experiments to ensure proper dispersion and stability of the copper chloride.

**Transwell Assays**

Tumor cells were seeded into the upper chamber with Matrigel matrix (Corning, diluted at 1:6 with RPMI 1640), and 600 μl of medium supplemented with 20% FBS plus Cu or not was added to the lower chambers. After 24-hour incubation, the cells on the lower surface were fixed with methanol for 15 min and stained with 0.5% crystal violet (Beyotime) at room temperature for 15 min.

**Cell Scratch Assay**

KYSE150 cells were seeded into 6-well plates for 24 hours and allowed to reach confluency as needed. The cells were scratched via a 200 μl pipette tip and washed 3 times with PBS before a medium containing Cu was added. RT at 12 Gy was delivered within 1 hour before scratching. The wounds were photographed at 0 h and 6 h. Gap closure was measured in 3 random regions of interest, and the average wound gap from those regions was calculated via ImageJ software (National Institutes of Health, USA). The percentage of wound-healing rate = (average width at 0 hours - average width at 6 hours)/average width at 0 hours × 100%.

**Western Blotting Analysis**

Total protein was extracted from the cells with RIPA lysis buffer (Solarbio) supplemented with a 1% protease inhibitor cocktail (Beyotime). Proteins were separated via SDS-PAGE and transferred onto a 0.45 μm polyvinylidene difluoride membrane (Millipore), followed by incubation overnight at 4℃ with the corresponding antibodies. The primary antibodies used in this study included antibodies against GLS2 (1:1000, ABclonal), DLST (1:1000, ABclonal), LIAS (1:1000, Proteintech), and β-actin (1:1000, Proteintech). HRP-conjugated affinipure goat anti-rabbit or anti-mouse IgG (Proteintech) was used. The supersensitive chromogenic reagent (PK10003, Proteintech) was used for the color reaction. The blots were imaged via a GE imager (Amersham Imager 600, GE Healthcare Life Sciences).

**RT-PCR**

Total RNA was extracted from cells via TRIzol reagent (Thermo Fisher Scientific) following the manufacturer’s guidelines, and cDNA synthesis was performed via the Prime Script^TM^ RT Reagent Kit (Takara, Ostu, Japan) following the manufacturer’s protocols. Reverse transcription polymerase chain reaction (RT-PCR) was performed via Hieff SYBR Green master mix (Yesen, China) on a QuantGene 9600 system (Bioer Technology, China). The $2^{\text{-∆∆Ct}}$ method was used for comparisons between groups. β-actin was used as an internal control. The primers used for qPCR were listed as follows:

Human GLS2 forward: 5’-GACCCAGGCATTCCGAAAGA-3’

Human GLS2 reverse: 5’-GGGATGTAGGCTGCCACTTT-3’

Human β-actin forward: 5’-GTGACGTTGACATCCGTAAAGA-3’

Human β-actin reverse: 5’-GCCGGACTCATCGTACTCC-3’

**RNA Interference (siRNA)**

KYSE150 cells were seeded in 6-well plates containing 2 ml of RPMI 1640 medium and cultured for 24 hours until they reached approximately 70% - 90% confluence. Subsequently, 20 pmol of siRNA agent targeting human GLS2 was diluted in 50 μL of complete culture medium and combined with 2 μL of EZ trans RNA agent (Shanghai Life-iLab Biotech Co., Ltd, China). After thorough mixing, the solution was incubated at room temperature for a duration of two minutes before being added to parental KYSE150 cells. Following an additional round of mixing, the cells were placed in a 37°C incubator under a 5% CO_2_ atmosphere, where they remained for a period of 24 - 48 hours. There was no need to replace the medium. At the end of this timeframe, RT-PCR analysis was conducted to evaluate the efficacy of the RNA transfection process. The sequence of the siRNA used was the same as that used in Suzuki et al.[22].

Human GLS2 siRNA1: 5′-GGACACATCGAAGTTGTTAAA-3′

Human GLS2 siRNA2: 5′-ATCAAGATGGACTGTAACAAA-3′

**Clonogenic Assay**

KYSE-150 cells were seeded in 6-well plates containing 2 ml of RPMI 1640 medium and incubated for 24 hours. The cells were then treated with Cu (at the IC30 concentration) along with ES and/or TTM (Selleckchem) for 24 hours, with the dosage of ES and TTM administered at a ratio of 1:1 relative to that of Cu. Following treatment, the cells were further cultured in complete medium for an additional period of 1 - 2 weeks. For the radiation group, cells were seeded as described above. After 24 hours, the plates were assigned to different groups on the basis of the radiation dose. Four hours post-Cu treatment, cells were subjected to single doses of radiation (2 Gy, 4 Gy, and 12 Gy) via a linear accelerator (Trilogy^TM^, Varian, USA). Then, the cells were returned to normal medium for 1 - 2 weeks. Colonies with more than 50 cells were counted under an EVOS^TM^ XL Core Imaging System (Thermo Fisher Scientific, USA). The visible colonies were fixed with methanol and stained with crystal violet. The survival fraction was defined as the ratio of treated cells to control cells.

**ROS Measurement**

A Reactive Oxygen Species Assay Kit (Beyotime) was used to detect the cellular ROS levels. Following the experimental protocol, the cells were treated with different drugs or radiation therapy. One hour after treatment, the DCFH-DA probe (diluted 1:1000 in serum-free culture medium) was added to the cells. After the culture medium was removed, 100 μl of the diluted DCFH-DA probe was added to each well of a black, clear-bottom 96-well plate (Beyotime, FCP965). The cells were incubated at 37°C in a cell incubator for 20 minutes. After incubation, the cells were washed three times with serum-free culture medium. Fluorescence was detected via a fluorescence spectrophotometer (excitation wavelength of 488 nm and emission wavelength of 525 nm; 1510, Thermo Fisher Scientific, USA). Typical images of ROS expression were taken with a laser confocal microscope (Axio Vert.A1, ZEISS)

**α-KG Concentration** **Measurement**

The α-KG concentration was measured using the α-KG Assay Kit from Solarbio, Beijing, China. Briefly, 1ml of extraction solution was added to 5×10^6^ cells (volume ratio: 5:1), the cells were disrupted via ultrasonication in an ice bath (power of 300 W, ultrasonication for 3 seconds, interval of 7 seconds, total duration of 3 minutes), and the mixture was subsequently centrifuged at 4°C and 12, 000 × g for 10 minutes. A total of 0.8ml of the supernatant was removed, 150ul of extraction solution 2 was slowly added, and the mixture was gently mixed by blowing until no bubbles formed. The mixture was then centrifuged again at 4°C and 12, 000 × g for 10 minutes, after which the supernatant was retained for testing. The UV spectrophotometer was preheated for 30 minutes, the wavelength was adjusted to 340 nm, and the UV spectrophotometer was zero with distilled water. In a 96-well UV plate, samples were added as follows: determination tube (sample 60 μl) / standard tube (standard substance 60 μl) / blank tube (distilled water 60 μl) + reagent one 110 μl + reagent two 10 μl + reagent three 10 μl; then, the mixture was heated at 37 °C for 5 minutes, reagent four 10 μl was added, mixed thoroughly, and the absorbance value A1 was measured at 20 seconds at 340 nm; then, the mixture was reacted for 5 minutes at 37°C, and the absorbance value A2 was measured at 5 minutes and 20 seconds. The α-KG content was subsequently calculated according to the formula in the instruction manual.

**α-KGDH activity Measurement**

Alpha-ketoglutarate dehydrogenase (α-KGDH) activity was determined via the α-KGDH Assay Kit from Solarbio, Beijing, China. Briefly, approximately 5×10^6^ cells were collected in a centrifuge tube, and 1ml of reagent one and 10 μl of reagent two were added and centrifuged at 4°C and 11, 000 × g for 10 minutes. The supernatant was retrieved. The spectrophotometer (Model 1510, Thermo Fisher Scientific) was preheated for 30 minutes. Transfer 200 μl of working solution into a 96-well UV plate and incubate at 37 °C for 5 minutes. Then, 8 μl of reagent eight and 12 μl of distilled water were added to the blank tube, or 12 μl of supernatant solution as extracted above was added to the measurement tube in order. A timer was used for 10 seconds, the samples were mixed thoroughly, and the absorbance value at 340 nm was measured immediately after 10 seconds. The mixture was subsequently incubated at 37 °C for an additional 2 minutes, and the absorbance value at 340 nm was recorded at 2 minutes and 10 seconds. Finally, α-KGDH activity was calculated according to the formula provided in the manual instructions.

**Succinate Rescue**

The KYSE150 cells were transduced with siGLS2. Then 2mM of monomethyl succinate (Me-succinate) was supplemented in cell culture media and subsequently added every 24h thereafter. The growth rates of cells were determined at 24, 48 and 72 hours using Alamar blue agents.

**Cell line-derived Xenograft Model**

Xenograft tumors were established by subcutaneously injecting KYSE-150 cells (1x10^6^ cells per mouse) into the right flanks of four- to six-week-old female nude mice (BALB/C), which were purchased from the Vital River Laboratory Animal Technology Co., Ltd. (Beijing, China). The mice were monitored daily for tumor growth, and the tumor volume was measured via digital calipers. Upon the tumor volume reached 50 mm^3^ - 100 mm^3^, the mice were randomly divided into several groups (n = 5 per group). Therapeutic agents were administered every 3 days for a total of 3 administrations via tail vein injection at a dosage of 3.5 mg/kg for both Cu and ES, and TTM was delivered intraperitoneally at a dosage of 10 mg/kg. For the groups receiving radiation therapy, radiation was delivered via a linear accelerator with a total dose of 12 Gy, delivered in 3 fractions of 4 Gy each over consecutive days, which was synchronized with the drug treatment. Tumor dimensions were gauged every 3 days post-inoculation with the aid of digital calipers, and the volume was calculated via the following formula: volume = (length/2) × width^2. The reagents used in this study are summarized in Table 2. All the animal studies were conducted in accordance with the Association for Assessment and Accreditation of Laboratory Animal Care guidelines and under the approval of the Institutional Animal Care and Use Committee of Shandong Provincial Hospital (No. 2023-014).

**Statistical Analysis**

Statistical analyses were carried out using Student’s *t*-test for pairwise comparisons between two groups and one-way analysis of variance (ANOVA) supplemented by post-hoc Tukey’s tests for multiple comparisons across more than two groups. All calculations were performed with SPSS version 29.0 (IBM, Chicago, IL, USA). All experimental data are presented as the Mean ± Standard deviation (SD) for bar graphs and the Mean ± SEM for line graphs from at least three independent experiments. Statistical significance was determined at **p* < 0.05, ***p* < 0.01, ****p* < 0.001, and *****p* < 0.0001.
